# Designing a Seasonal Acclimation Study Presents Challenges and Opportunities

**DOI:** 10.1093/iob/obac016

**Published:** 2022-04-28

**Authors:** Raymond B Huey, Lauren B Buckley

**Affiliations:** Department of Biology, University of Washington, Seattle, WA 98195, USA; Department of Biology, University of Washington, Seattle, WA 98195, USA

## Abstract

Organisms living in seasonal environments often adjust physiological capacities and sensitivities in response to (or in anticipation of) environment shifts. Such physiological and morphological adjustments (“acclimation” and related terms) inspire opportunities to explore the mechanistic bases underlying these adjustments, to detect cues inducing adjustments, and to elucidate their ecological and evolutionary consequences. Seasonal adjustments (“seasonal acclimation”) can be detected either by measuring physiological capacities and sensitivities of organisms retrieved directly from nature (or outdoor enclosures) in different seasons or less directly by rearing and measuring organisms maintained in the laboratory under conditions that attempt to mimic or track natural ones. But mimicking natural conditions in the laboratory is challenging—doing so requires prior natural-history knowledge of ecologically relevant body temperature cycles, photoperiods, food rations, social environments, among other variables. We argue that traditional laboratory-based conditions usually fail to approximate natural seasonal conditions (temperature, photoperiod, food, “lockdown”). Consequently, whether the resulting acclimation shifts correctly approximate those in nature is uncertain, and sometimes is dubious. We argue that background natural history information provides opportunities to design acclimation protocols that are not only more ecologically relevant, but also serve as templates for testing the validity of traditional protocols. Finally, we suggest several best practices to help enhance ecological realism.

## Chinese

生活在季節性環境中的生物經常調整其生理能力和生理敏感性以響應(或預判)環境變化。這些生理和形態上的調整(“馴化”和相關術語)啟發研究者去探索背後的機制、檢測誘導產生這些調整的信号並闡明其生態和進化後果。季節性調整(“季節馴化”)可以通過測量在不同季節從野外(或室外圍欄)採集的生物體的生理能力和生理敏感性來檢測, 也可以間接地通過測量飼養在模擬自然條件的實驗室環境中的生物體來檢測。但在實驗室中模擬自然條件存在諸多困難—需要事先了解與物種生態相關的體溫循環、光週期、食物配給、社會環境等博物學知識。我們認為, 傳統的實驗室條件通常無法準確模擬野外不同季節的環境條件(溫度、光週期、食物、“封鎖”)。因此, 在這些實驗室條件下產生的馴化響應是否接近野外真實發生的情況存在不確定性, 甚至是非常可疑的。我們認為, 了解博物學背景信息有助於设计出更加具有生态相关性的驯化流程, 并应用这些标准化的流程去检验传统驯化流程的缺陷。最後, 我們提出了一些有助於增強生態現實性的方案。

## French

Les organismes vivant dans des environnements saisonniers ajustent souvent leurs capacités et leurs sensibilités physiologiques en réponse (ou en prévision de) aux changements environnementaux. De tels ajustements physiologiques et morphologiques (« acclimatation » et termes apparentés) offrent l“opportunité d'explorer les mécanismes sous-jacents à ces ajustements, de détecter les indices qui les induisent et d’élucider leurs conséquences écologiques et évolutives. Les ajustements saisonniers (“acclimatation saisonnière”) peuvent être détectés soit en mesurant les capacités physiologiques et les sensibilités d'organismes prélevés directement dans la nature (ou dans des enclos extérieurs) à différentes saisons, soit de manière moins directe en élevant et en mesurant des organismes maintenus en laboratoire dans des conditions qui tentent d”imiter ou de suivre les conditions naturelles. Mais il est difficile de reproduire les conditions naturelles en laboratoire car il faut pour cela connaître les cycles de température corporelle, la photopériode, le régime alimentaire, les environnements sociaux, entre autres variables pertinentes d'un point de vue écologique. Nous argumentons que les conditions traditionnellement utilisées en laboratoire ne parviennent généralement pas à se rapprocher des conditions saisonnières naturelles (température, photopériode, nourriture, « confinement »). Par conséquent, il n“est pas certain, et parfois douteux, que les écarts d”acclimatation qui en résultent se rapprochent correctement de ceux de la nature. Nous soutenons que les informations de base sur l“histoire naturelle offrent la possibilité de concevoir des protocoles d”acclimatation qui sont non seulement plus pertinents sur le plan écologique, mais servent également de modèles pour tester la validité des protocoles traditionnels. Enfin, nous suggérons plusieurs bonnes pratiques pour aider à améliorer le réalisme écologique.

## German

Organismen, die in saisonalen Umgebungen leben, passen häufig ihre physiologischen Fähigkeiten und ihre Sensitivität als Reaktion auf (oder in Erwartung von) Umweltveränderungen an. Solche physiologischen und morphologischen Anpassungen („Akklimatisierung“ und verwandte Begriffe) bieten die Möglichkeit, die diesen Anpassungen zugrunde liegenden mechanistischen Grundlagen zu erforschen, Reize zu erkennen, die Anpassungen auslösen, und ökologische und evolutionäre Konsequenzen aufzuklären. Saisonale Anpassungen („saisonale Akklimatisierung“) können entweder durch Messung der physiologischen Kapazitäten und Sensitivität von Organismen, die zu verschiedenen Jahreszeiten direkt aus der Natur (oder Außengehegen) entnommen wurden, oder weniger direkt durch Aufzucht und Messung von Organismen, die im Labor unter Bedingungen gehalten werden, die eine Nachahmung oder Nachverfolgung der natürlichen Bedingungen anstrebt, detektiert werden. Allerdings ist die Nachahmung natürlicher Bedingungen im Labor eine Herausforderung—dies erfordert unter anderem Kenntnisse über ökologisch relevante Körpertemperaturzyklen, Photoperioden, Nahrungsrationen, sowie das soziale Umfeld. Wir argumentieren, dass traditionelle Laborbedingungen normalerweise nicht den natürlichen saisonalen Bedingungen entsprechen (Temperatur, Photoperiode, Nahrung, „Lockdown“). Ob die resultierenden Akklimatisierungsverschiebungen denen in der Natur genau entsprechen, ist daher ungewiss und manchmal zweifelhaft. Wir argumentieren, dass naturgeschichtliche Hintergrundinformationen Möglichkeiten bieten, Akklimatisierungsprotokolle zu entwerfen, die nicht nur ökologisch relevanter sind, sondern auch als Vorlagen zum Testen der Gültigkeit traditioneller Protokolle dienen können. Abschließend schlagen wir mehrere Best Practices vor, um den ökologischen Realismus zu verbessern.

## Spanish

Los organismos que viven en ambientes estacionales pueden ajustar sus capacidades y sensibilidades fisiológicas en respuesta (o en anticipación) a cambios ambientales. Estos ajustes fisiológicos y morfológicos (“aclimatación” y términos afines) dan la oportunidad para explorar el mecanismo que subyace a estos ajustes, también para detectar las señales que inducen tales ajustes y finalmente para dilucidar sus consecuencias ecológicas y evolutivas. Los ajustes estacionales (“aclimatación estacional”) se pueden detectar midiendo las capacidades y sensibilidades fisiológicas de los organismos, ya sea en especímenes extraídos directamente de la naturaleza (o recintos al aire libre) en diferentes estaciones, como también, de una manera menos directa, en especímenes criados y mantenidos en el laboratorio bajo condiciones que simulan las condiciones naturales y sus cambios estacionales. Sin embargo, esta simulación en el laboratorio es un desafío; hacerlo requiere un conocimiento previo de la historia natural de los ciclos de temperatura corporal, los fotoperíodos, las raciones de alimentos, los entornos sociales, entre otras variables ecológicamente relevantes. Argumentamos que las condiciones tradicionales de laboratorio generalmente no se aproximan a las condiciones estacionales naturales (temperatura, fotoperíodo, comida, “bloqueo”). En consecuencia, es incierto y, a veces, dudoso si los cambios de aclimatación resultantes se aproximan correctamente a los de la naturaleza. Así también, la información de antecedentes de la historia natural brinda oportunidades para diseñar protocolos de aclimatación que no solo son más relevantes desde el punto de vista ecológico, sino que también sirven como plantillas para probar la validez de los protocolos tradicionales. Finalmente, sugerimos varias mejoras prácticas que pueden ayudar a lograr un realismo ecológico optimizado en las simulaciones de laboratorio.

## Introduction

*“… a frog or a toad is by no means the same thing in summer as in winter.”*

Claude Bernard, 1865 (1949 edition)

Seasonality is a fact of nature for almost all terrestrial organisms, especially those at higher latitudes and altitudes. In anticipation of—or in reaction to—such seasonal environmental variation, organisms often adjust their behavior, physiological capacities, and environmental sensitivities via internal physiological adjustments that are variously called acclimation, acclimatization, or phenotypic plasticity (Levins 1968; Sultan 2015). Given that 1-½ centuries have elapsed since Claude Bernard pioneered studies of seasonal physiological plasticity in ectotherms (Bernard 1949), a newcomer to this field might expect that associated experimental protocols would be well established and long validated. However, we argue here that common laboratory protocols (especially those involving acute shifts of temperature or photoperiod) are in fact ecologically dubious, sometimes damaging (Jensen et al. 2017), and have rarely been validated against phenotypic shifts in nature. Such issues weaken attempts to use laboratory results to help predict phenotypic responses to seasonal or climate change (Angilletta 2009, p. 154; Somero 2010; Seebacher et al. 2015; Gunderson et al. 2016; Buckley & Kingsolver 2019; Gibert et al. 2019; Terblanche & Hoffmann 2020). We suggest that seasonal natural history information can guide development of protocols that may improve the eco-evolutionary and physiological relevance of seasonal plasticity experiments. We focus on animal ectotherms, but many ideas apply to plants and endotherms.

### Our perspective on terminology

Physiologists often restrict “acclimatization” to physiological shifts occurring in nature and restrict “acclimation” to investigator-driven shifts in the laboratory, typically involving controlled manipulations of one or a few environmental variables (reviewed in Somero et al. 2017, p. 12–13). Unfortunately, these distinct terms divert focus from the physiological responses themselves to the venues of study. The distinction has persisted because field biologists have rarely examined seasonal changes in environments and in physiology in nature (information that laboratory physiologists need to design ecologically relevant experiments) and because few laboratory facilities were capable of controlling dynamic shifts in multiple environmental factors. Adjusting one or a few factors does achieve experimental control and reproducibility but sacrifices ecological realism.

Here, we use “acclimation” as an umbrella term for studies of seasonal responses. We argue that the traditional distinction (acclimation versus acclimatization) has become both antiquated and counterproductive in the context of seasonal plasticity. It is antiquated because ecology and physiology are mutually dependent and represent mutually informative levels of biological analysis (Bartholomew 2005). It is antiquated because contemporary environmental facilities are increasingly capable of complex environmental manipulations (below). It is counterproductive because it reinforces separations between ecology and physiology as well as between descriptive and experimental approaches. Accordingly, we will use “acclimation” here to refer to both field and laboratory responses to seasonal change.

In addition, we use seasonal acclimation for species with multi-generations per year, even though acclimation is traditionally restricted for individuals, not generations. Species with a sequence of generations across seasons offer opportunities to explore between-generation causes, mechanisms, and ecological consequences of seasonal changes in phenotypes (Rudman et al. 2022).

Our paper is a part of a long-standing effort by many to push for greater interactions between field biologists, who now can monitor and simulate seasonal changes in phenotypes and environmental factors, and laboratory biologists, who can design ecologically realistic, controlled, and multi-factorial experiments (Bartholomew 1964; Chown & Gaston 1999; Loeschcke & Hoffmann 2007; Kearney et al. 2014; Gunderson et al. 2016; Somero et al. 2017; Denny 2018; Rudman et al. 2022, p. 13).

### General goals for seasonal acclimation studies

We begin by conceptualizing three individual but complementary goals of a hypothetical study of seasonal plasticity of trait(s) in an arbitrary ectotherm. First, quantify seasonal variation in, for example, the thermal sensitivity of trait performance or capacity (independent of short-term hardening responses, see Zhang et al. 2021). Second, probe the underlying environmental, behavioral, and physiological cues and drivers of those seasonal shifts (and interactions). Third, elucidate the ecological and evolutionary consequences of seasonal shifts (Kingsolver & Wiernasz 1991; Loeschcke & Hoffmann 2007; Somero 2010; Terblanche & Hoffmann 2020; Rudman et al. 2022). However, the techniques necessary to evaluate those consequences are beyond the scope of this paper and will not be discussed here.

Achieving the first goal of describing acclimation patterns would seem relatively easy, and three general methods can be used.

1. One can directly—and unambiguously—quantify seasonal patterns in physiology and morphology by collecting organisms from nature in each season and quickly measuring their trait values and sensitivities.
2. One can release organisms into semi-realistic enclosures in nature and then periodically extract individuals for measurements. This approach is logistically appealing because retrieving individuals from enclosures is often easier than from nature. In any case, these first two methods both yield “realized” acclimation (acclimatization) patterns.
3. Finally, controlled laboratory experiments can be designed to induce seasonal responses that approximate those of organisms in nature. However, because seasonal changes in environments and physiological activities are complex, multi-factor manipulations are required but can be daunting. Consider an experiment with three different temperature cycles, three photoperiod cycles, and three food regimes. When faced with all the critical variations on this approach (each with main and interactive effects) plus replication, many researchers will quickly conclude multi-factorial approaches are intractable for most organisms (see especially Fig. 6 in Boyd et al. 2018; but see Porter et al. 1984; Singh et al. 2020).

Here, we address key challenges to designing laboratory acclimation protocols that are intended to induce physiological responses that approximate natural ones (goal one, above). Our suggestions are guided by our experiences with terrestrial ectotherms (lizards, insects) but should hold for other mobile ectotherms living in spatially heterogeneous environments. We make no attempt to be exhaustive but rather focus on four factors that are common to most acclimation studies (body temperature, photoperiod, food ration, and “social distancing and lockdown”). Other physical factors can of course be relevant (e.g., barometric pressure for altitude acclimation; pH, salinity, and hypoxia in aquatic systems). We will describe traditional protocols for manipulating each of these factors, then argue that such manipulations generally bear little resemblance to the shifting and fluctuating environments experienced by organisms in nature (see Angilletta 2009, p. 154), and suggest “best practices” to enhance realism.

### Key problems with seasonal-acclimation experiments

#### Laboratory conditions are not ecologically relevant

Even though experimental conditions should attempt to mimic ones in nature, experimental conditions (e.g., temperature cycles, photoperiods) are often not ecologically relevant (Schou et al. 2015). Importantly, specific protocols sometimes generate different responses and experimental artifacts (see Fig. 2 in Rohr et al. 2018; Terblanche & Hoffmann 2020).

Researchers sometimes guess at conditions that seem ecologically relevant, make choices for experimental convenience (e.g., constant temperature treatments), manipulate only one or a few environmental variables, and ignore natural environmental, developmental, and cross-generational variation (Crill et al. 1996; Bradshaw & Holzapfel 2006; Robolledo et al. 2021). Few workers have tested whether targeted laboratory variables such as temperature are in fact “key factors” in nature (Ives & Gilchrist 1993; Angilletta 2009). For example, researchers working with lizards have—for many decades—manipulated only temperature. However, the importance of moisture is increasingly appreciated (Clusella-Trullas et al. 2011; Kearney et al. 2018; Rozen-Rechels et al. 2021). In general, multi-factor experiments will be required to understand seasonal acclimation responses (Danks 2007; Gunderson et al. 2016; Somero et al. 2017, p. 13; Terblanche & Hoffmann 2020). Furthermore, few studies consider the influence of biotic interactions (Davis et al. 1998; Nespolo et al. 2022) or the magnitude of individual and genotypic variation (Dowd et al. 2015; Terblanche & Hoffmann 2020; Messerman & Leal 2021; Seebacher & Little 2021; Winterová & Gvoždík 2021).

A less arbitrary approach is to use natural history data as guides for laboratory conditions (Bradshaw & Holzapfel 2001; Fangue & Bennett 2003; Niehaus, Angilletta, et al. 2012; Basson & Clusella-Trullas 2015; Toxopeus et al. 2019). Or, as Lewontin wryly noted (2000, p. 54), “*If one wants to know what the environment of an organism is, one must ask the organism*.” We explore this view below.

#### Laboratory conditions block behavioral adjustments

In nature, animals are not only affected by changes in their environment but also actively choose their own environment: “*Organisms are both the subjects and the objects of evolutio*n” (Levins & Lewontin 1985, p. 275). In other words, “. . .*the histories of both environment and organism are functions of both environment and organism*” (Lewontin 2000, p. 101). In contrast, laboratory environments are physically restrictive and force animals to passively accept conditions chosen by the experimenter. This gives experimental control but prevents animals from making behavioral adjustments (in exposure time, operative environment, and social behaviors) or moving about, as they would do in nature (Hadamová & Gvoždík 2011; Salachan et al. 2020). Such constraints on behavior potentially mask natural seasonal responses (Brankatschk et al. 2018; Salachan et al. 2020) and potentially induce stress or pathologies.

Consider a photoperiod experiment in which individuals will be forced to experience a specific photoperiod, but that might not do so if given a choice. Experiments with hatchling lizards illustrate variation in voluntary exposure to light. For example, hatchling lizards of a high-elevation species of *Sceloporus* voluntary exposed themselves to a heat lamp for shorter periods each day than did hatchlings from a high-elevation species (Sinervo & Adolph 1989), as did as populations of a high elevation species in the field (Sinervo 1990). Would forced exposure to long days induce stress in individuals that would normally retreat in the field?

Consider the “habitat matching” model (see Fig. 1 in Jacob et al. 2015), in which unconstrained individuals can disperse to find and settle in habitats suitable for their particular phenotype (e.g., if males and females have different thermal preferences, Lailvaux 2007). But in a fixed acclimation treatment, all phenotypes are forced to experience specified conditions, even if some individuals would have dispersed away from such conditions in nature. Would that induce stress in forcibly “mis-matched” individuals? We see behavioral restriction in the laboratory as a potential confound in acclimation studies, whether seasonal or not, and needing study.

**Fig. 1.**
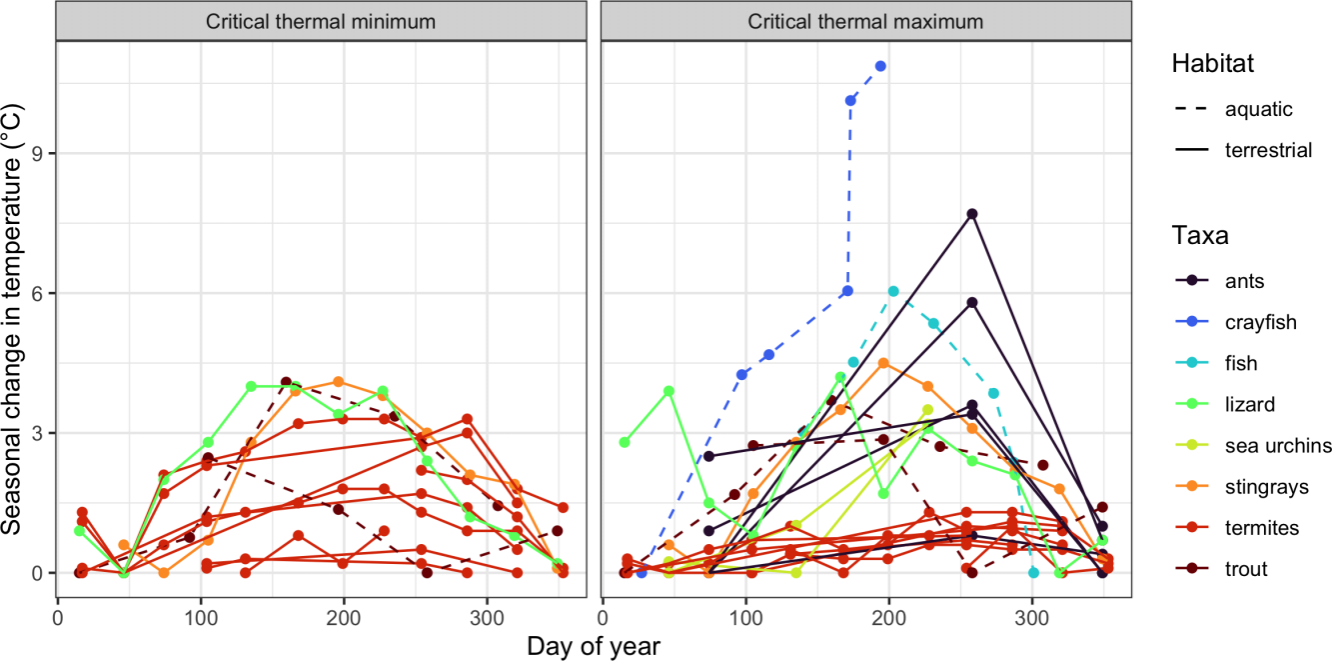
Examples of change in *CT*_max_ and in *CT*_min_ (difference from lowest seasonal value) in animals collected in nature over the seasons. Some species (e.g., termites) show little seasonal change, but other show marked change. Seasonal studies provide a realized baseline for validating laboratory estimates of critical temperatures (or other traits). References: (Mundahl 1989; Fangue and Bennett 2003; Hu and Appel 2004; Sharma et al. 2015; Sherman 2015; Domínguez-Guerrero et al. 2019; Kamalam et al. 2019; Bujan et al. 2020; Leclair et al. 2020).

### Seasonal acclimation in the field

Collecting and quickly measuring animals from the field (or from enclosures) in different seasons is the “gold standard” for assaying directions and magnitudes of realized seasonal acclimation. Moreover, such field studies are necessary for validation studies that attempt to evaluate whether laboratory acclimation protocols in fact yield ecologically relevant responses.

Examples of such field studies (Fig. 1) are shown for critical thermal maximum and minimum (*CT*_max_, *CT*_min_ — upper or lower thermal indices of performance, respectively) (Bennett et al. 2018). These studies show elevated heat tolerance in summer and increased cold tolerance in winter, but also show considerable interspecific variation in the magnitude of “realized” seasonal responses (Fig. 1).

Of course, obtaining animals in nature in some seasons can be challenging, dangerous, or even impossible. Furthermore, the results are descriptive (but see below) and apply only to local populations and conditions; and they do not illuminate whether observed seasonal responses reflect individual, cross-generation, or genetic differences (Stone et al. 2020). But they do provide a critical baseline.

### Factors often manipulated in seasonal-acclimation experiments

#### Body temperature

**Fig. 2:**
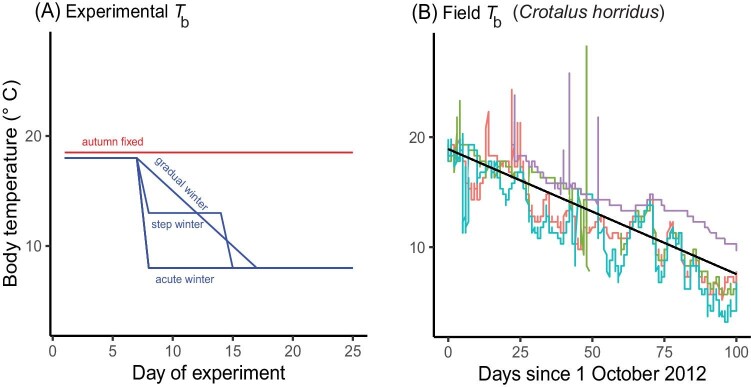
(A) Typical experimental protocols involving body temperature in seasonal acclimation experiments (autumn to winter). Here a 10 °C drop from “autumn” temperatures is achieved in a maximum of 10 days. (B) Realized Tb shifts for four timber rattlesnakes in Tennessee in autumn and early winter (data from Nordberg and Cobb 2017). Here a 10 °C drop took about 89 days (based on black regression line for all points), much longer than in laboratory experiments in A. Note that individual snakes (colors) had different Tb trajectories; and some had marked diel cycles of Tb.

Body temperature of most terrestrial ectotherms varies daily and seasonally (Clusella-Trullas & Chown 2014; Nordberg & Cobb 2017), even in the tropics (Christian et al. 1983; Hertz 1992; Salazar et al. 2019). Yet acclimation treatments often use fixed temperature treatments with rapid transitions between treatments (c.f. Angilletta 2009; see Supplement in Gunderson & Stillman 2015; Terblanche & Hoffmann 2020) and may confound seasonal and “heat-hardening” (i.e., brief exposure to sub-lethal temperature) responses (Loeschcke & Hoffmann 2007; Phillips et al. 2015). Seasonal acclimation studies are more likely to use gradual temperature transitions than are studies addressing climate change issues (Gunderson & Stillman 2015; Seebacher et al. 2015), but the rates are still abnormally fast (Table S1). For example, animals might be transferred acutely from a fixed and warm baseline temperature regime (“warm season”) to a “cool season” one (Fig. 2A). Sometimes, however, an animal's temperature is stepped down over several weeks (e.g., −5°C every six days, *Thamnophis marcianus*, Holden et al. 2021) or is lowered more gradually (e.g., 1°C per day, *Tachydromus* spp., Huang & Tu 2008)(Fig. 2A).

In the above examples, *T*_b_ will drop by 10°C in a maximum of only 10 days. In contrast, the *T*_b_ of timber rattlesnakes (*Crotalus horridus*) in retreats in Tennessee (Nordberg & Cobb 2017) took three months to drop about 10°C; and *T*_b_ dropped erratically, differed among individuals, and included daily cycles (Fig. 2B)! Thus, gradual or step drops typically used in the laboratory can be much faster than are those in nature, while ignoring diel and stochastic variation (cf. Sinclair 2001; Dillon & Lozier 2019; Sørensen et al. 2020). Examples of studies that used more realistic shifts include Bradshaw and Holzapfel (1989), Costanzo et al. (2000), Neihaus et al. (2012), and Toxopeus et al. (2019). Natural *T*_b_ trajectories can be obtained via radio-telemetry, attached/implanted data loggers (Cobb & Peterson 2008; Davis et al. 2008) (Fig. 2B), or biophysical simulations (Buckley 2008; Kearney, Deutscher, et al. 2020).

Do abnormally fast drops and short acclimation durations found in most laboratory experiments (Table S1) allow sufficient time for normal acclimation adjustments (Angilletta 2009), or might they even be pathological? This is hard to predict, but many physiological responses are sensitive to rates and duration of temperature change (Nilsson-Örtman & Johansson 2007; Terblanche et al. 2007; Jørgensen et al. 2019). Also, some responses require weeks of acclimation to be manifest (Toxopeus et al. 2019). Consequently, using natural rates of temperature change in the laboratory may be the safest way to generate realistic responses to seasonal acclimation. Of course, “long and slow” acclimation might deplete energy reserves or induce cold damage (Sinclair 2015). Even so, that could be appropriate if “long and slow” is what happens in nature (Fig. 2B).

Thermal regimes used in laboratory acclimation experiments generally do not incorporate diel individual, stochastic, microhabitat and day-to-day variation in *T*_b_ (Bradshaw et al. 2004; Niehaus, Wilson, et al. 2012) (e.g., Table S1, Fig. 2B). However, individual differences in environmental exposure can be pronounced in nature (Denny 2018; Carlson et al. 2021), especially during seasonal transitions (Taylor et al. 2004; Nordberg & Cobb 2017), except deep in the soil (fig. 4 in Huey, Ma, et al. 2021). Such differences can have marked physiological impacts (Clarke & Zani 2012; Niehaus, Angilletta, et al. 2012; Dowd et al. 2015; Wiebler et al. 2017).

Whether suppression of natural variation in *T*_b_ biases acclimation responses is rarely studied (but see Estay et al. 2010; Hadamová & Gvoždík 2011; Niehaus, Angilletta, et al. 2012). Over a half century ago, Wilhoft (1958) showed that fence lizards (*Sceloporus occidentalis*) had elevated death rates if maintained at their normal activity temperature (34–35°C) for several weeks. Subsequent studies demonstrate that constant-temperature treatments may induce pathologies and alter performance profiles (Schulte et al. 2011; Colinet et al. 2015; Cavieres et al. 2016). Persistent temperature exposures (Rezende et al. 2014; Kingsolver & Woods 2016; Jørgensen et al. 2019) and repeated exposures can be stressful (Marshall & Sinclair 2015).

**Fig. 3:**
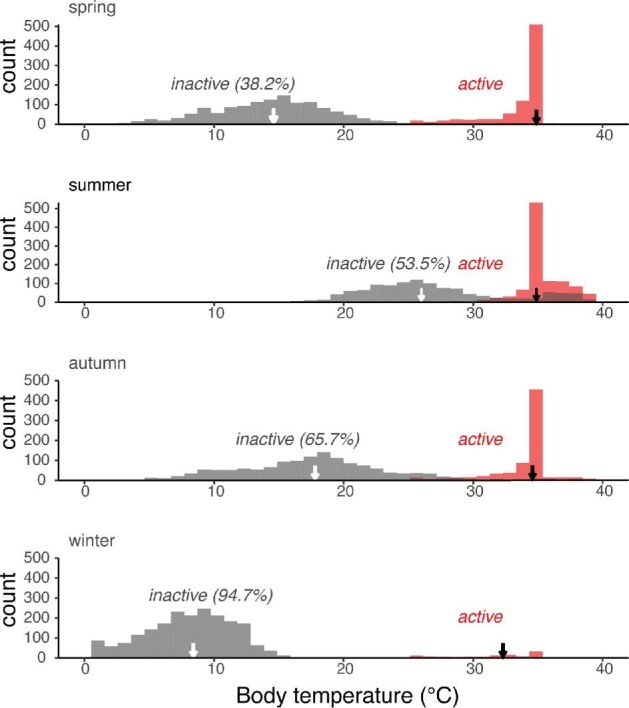
Simulated body temperature distributions of a lizard (10 g) at Ford Dry Lake, CA (see online supplement for methods). Red = active (basking, foraging) lizards, gray = inactive lizards. The arrows indicate median *T*_b_ of inactive and of inactive animals by season, and the percentage of all animals that were inactive is indicated. Note that median *T*_b_ of active animals is rather independent of season, whereas that of inactive animals drops markedly cool seasons. Note also that the percentage of animals that are inactive shifts dramatically among seasons.

Diel and day-to-day variation in *T*_b_ during dormancy can be marked in species that are intermittently active on warm winter days, as *T*_b_ jumps during such activity (Fig. 2B). A simulated example is shown in Fig. 3, which plots histograms of *T*_b_ (by activity status) for summer and winter. Three patterns are striking. First, *T*_b_ distributions are bimodal within seasons, and the median *T*_b_ of active individuals is much warmer than that of inactive animals. Second, the median *T*_b_ of active individuals (black arrows) changes very little among seasons, whereas the median *T*_b_ of inactive individuals (white arrows) shifts dramatically. Third, the relative areas under the active versus inactive modes also shifts seasonally. For example, 53.5% of all hourly *T*_b_ are from inactive animals in summer, but 94.7% are from inactive individuals in winter. Thus, a realistic acclimation *T*_b_ profile for this simulated animal will require diel shifts in mean inactive *T*_b_ (less so in active *T*_b_) and in the relative proportion of active versus inactive *T*_b_ by season. In an early example that considered such seasonal differences, Tsuji (1988) exposed lizards to 12 h at 35°C and 12 h at 16°C for summer conditions, but then used 6 h at 35°C and 18 h at 10°C for autumn conditions. Similarly, Zani (2012) gradually shifting photoperiods and thermoperiods for the lizard *Uta stansburiana*.

Incorporating a daily temperature cycle may be important (Bradshaw 1980; Brakefield & Mazzotta 1995; Hadamová & Gvoždík 2011; Colinet et al. 2015; Kingsolver et al. 2020) not only to reduce stress (above), but also because *T*_b_ has non-linear effects on physiology (Colinet et al. 2015). Seasonal variation in the magnitude of daily cycles in *T*_b_ in nature can be substantial (Bradshaw et al. 2004; Basson & Clusella-Trullas 2015). For simulated data in Fig. 3, the median daily range in *T*_b_ is varies three-fold among seasons [21.0°C (spring), 15.3°C (summer), 17.2°C (autumn), and 6.3°C winter)].

Other complications involving *T*_b_ regimes can be raised. In many ectotherms, *T*_b_ changes during ontogeny, as different developmental stages may live in different microenvironment, occur at different times of year, or have different tolerances (Zani et al. 2005; Kingsolver et al. 2011; Potter et al. 2013). Moreover, developmental and cross-generational effects can alter the temperature dependence of performance (Gilchrist & Huey 2001; Cavieres et al. 2019; Rebolledo et al. 2021). Thus, a seasonal acclimation study may need different thermal (and photoperiod) regimes for each developmental stages, and especially for seasonal acclimation in multi-voltine species, where different generations experience different conditions (Kingsolver et al. 2011; Sørensen et al. 2016; Terblanche & Hoffmann 2020). Also, individual and landscape variation in natural *T*_b_ profiles (Dowd et al. 2015) is expected (e.g., Fig. 2B), but whether such variation in *T*_b_ (e.g., Fig. 2B) often alters acclimation responses remains to be determined. It can affect overwinter survival and reproduction (Bradshaw & Holzapfel 1991; Otero et al. 2015).

As noted above, animals in environmental chambers typically have no opportunity for behavioral adjustments but are “force-fed” specific *T*_b_ profiles and simplified environments. Might such constraints on behavioral induce stress or alter acclimation patterns? In general, we suspect so (Bartholomew 1964; Glanville & Seebacher 2006; Jiménez-Padilla et al. 2020). Indeed, thermal preference of *Drosophila melanogaster* shifted with forced acclimation, but not when flies were reared in heterogeneous environment where they could behaviorally thermoregulate (Salachan et al. 2020).

For animals with multiple generations per year, winter and summer captured individuals in nature may be somewhat genetically different—a consequence of seasonal selection (Dobzhansky 1948; Rudman et al. 2022). Copepods (*Acartia* spp.) collected in summer were genetically more heat tolerant than those collected in winter, but had weaker acclimation responses (Sasaki & Dam 2020). Thus, an acclimation study based on a single cohort (e.g., summer collected) might yield misleading predictions of realized phenotypic patterns in winter.

*Best practices*.—We encourage laboratory studies that use ecologically relevant shifts in temperature, even though this will greatly lengthen the duration of experiments well beyond those of traditional ones (Table S1). Deciding on an “ecologically realistic” temperature profile will be challenging, given individual, microhabitat, and yearly variation (see Fig. 2B). Especially interesting will be validation studies that compare responses from traditional temperature exposures (fast, acute drops, no diurnal variation) versus those from ecologically realistic ones (Fig. 2B, 3) or that compare field with lab responses.

#### Photoperiod

Photoperiod is often the dominant environmental cue regulating observed seasonal shifts (Bradshaw & Holzapfel 2007) and can affect physiological tolerance (but see Moghadam et al. 2019; Toxopeus et al. 2019). For example, diel shifts in heat tolerance in *Drosophila buzzatii* are controlled by a physiological clock (Sørensen & Loeschcke 2002). Interestingly, freeze tolerance in the cricket *Gryllus veletis* requires shifts in both *T*_b_ and photoperiod (Toxopeus et al. 2019); and nymphal development in the cricket Modicogryllus siamensis depends on both photoperiod and temperatures pathways (Miki et al. 2020). Surprisingly, photoperiod is not adjusted in many seasonal experiments (Table S1), even though seasonally inappropriate photoperiods can cause major declines in performance or fitness (Bradshaw et al. 2004; MacLean & Gilchrist 2019; Le Roy & Seebacher 2020). Responses can depend not only on the length of the photoperiod, but also on the rate and direction of change of photoperiod (Norling 2018).

**Fig. 4:**
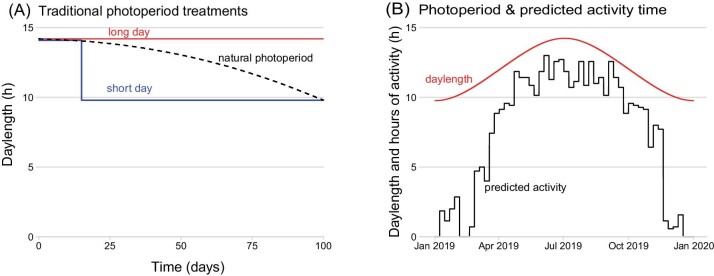
(A) Traditional photoperiod treatment of 14.2 h for summer versus 9.8 h for winter at Ford Dry Lake, For Review Only California, with an abrupt shift in photoperiod. (B) Time series of local photoperiod (red line) and potential exposure time (black line) of a simulated lizard over the year at Ford Dry Lake (see text). Note that predicted hours of exposure for this lizard was markedly lower than the actual day length, especially in winter. Thus, use of an acclimation photoperiod of 9.8 h for winter (A) may grossly overestimate the photoperiod perceived (B) by the animal.

A common protocol involves a rapid shift in photoperiod from long day (summer) to short day (winter) (Fig. 4A). Less commonly, photoperiod is adjusted gradually to match local photoperiod (Fig. 4A, Bradshaw & Holzapfel 1989; Toxopeus et al. 2019). However, acute or step shifts in photoperiod are more common, especially in older studies (see Norling 2018), when frequent adjustment of photoperiod was logistically challenging.

A priori, one might think that adjusting laboratory photoperiods to match natural ones at a given field site would be easy, at least if programmable environmental chambers are available. Note, however, the direct use of local photoperiods in acclimation experiments makes two implicit assumptions: (1) that nearby mountains are not delaying local sunrise or accelerating local sunset (Kearney, Gillingham, et al. 2020), and (2) that organisms in nature are fully exposed to and perceive the local photoperiod (Danks 2007). In reality, local photoperiod will approximate the realized photoperiod only for organisms that live in a flat and open landscape, that are always above ground, and that are always fully exposed to the sky. Few terrestrial organisms (other than some plants and birds) probably fit this bill. Whether this matters to plastic responses is unclear (see Bradshaw & Phillips 1980).

Most animals—whether diurnal or nocturnal—have restricted activity times, as regulation of above-ground activity time is the key behavioral adjustment that many terrestrial ectotherms use to regulate *T*_b_ (Stevenson 1985). In many reptiles, above-ground activity occupies a surprisingly small fraction of the year (Fig. 4 in Davis & DeNardo 2010; Table VII in Huey 1982). For example, desert tortoises (*Gopherus agassizii*) are above ground only 3% of the year (Marlow 1979), but whether they perceive light when underground is unclear. For inactive animals inside fully dark retreats, realized exposure time may be less—sometimes substantially less—than the local photoperiod (Kerr et al. 2004; Davis & DeNardo 2010). Furthermore, animals overwintering inside dark retreats (or in the Arctic) throughout winter will experience a 0:24 L: D photoperiod—obviously, no light exposure at all (Williams et al. 2016)! Similarly, aquatic ectotherms at depth may experience very dim or no natural light (Filatova et al. 2019). Local photoperiods can thus be a red herring in seasonal acclimation experiments and possibly confound seasonal responses.

To simulate how voluntary behavioral restriction can influence realized exposure time, we used NicheMapR (Kearney & Porter 2020) to estimate photoperiod as well as predict realized exposure time of a 10-g lizard at Ford Dry Lake, CA in 2019 (parameter values in online supplement). Over the year, local photoperiod varied from 9.8 to 14.2 h per day, and an acute acclimation shift using these photoperiods is shown in Fig. 4A. However, variation in predicted hours of exposure varied from 0 to 13 h/day, not 9.8 to 14.2 (Fig. 4B). In summer months, the difference between the median local photoperiod (14.0 h) and the median exposure time (11.4 h) was only 2.6 h (Fig. 4B); but in winter months, the median local photoperiod (10 h) was 9 h longer than the median exposure time (0.7 h). Moreover, simulated lizards in winter were completely inactive in some weeks, while active in others (Fig. 4B).

Are observed winter acclimation patterns sensitive to whether an experiment uses a local, mid-winter photoperiod (e.g., 9.7:14.3 L: D) or a predicted exposure-time one (e.g., 0.7:23.3 L: D)? Similarly, are acclimation patterns sensitive to whether ectotherms are intermittently active in winter (Nordberg & Cobb 2016; Huey, Miles, et al. 2021), and thus to whether they intermittently experience daylight? We know of no study with ectotherms that directly evaluates these issues, but brief exposures to elevated temperatures can potentially be physiologically beneficial (see Huey, Ma, et al. 2021, p. 181). In addition, physiological responses and even longevity can be sensitive to diel cycles in the intensity and spectral pattern of daylight (Shen & Tower 2019) as well as to dawn-dusk transitions (Bradshaw & Phillips 1980).

Natural history adds further complications. Consider the appropriate photoperiod for winter at mid-latitudes. In nature, a lizard overwintering a few centimeters in the soil experiences constant darkness, but one wedged in a nearby rock crevice might receive dim light cues. Also, *Uta stansburiana* lizards in eastern Oregon emerge from rock crevices and bask on sunny days even in mid-winter (P. Zani, personal communication), and such exposures will affect their *T*_b_ as well as their realized photoperiod. Are winter-acclimation responses of ectotherms sensitive to the interaction between light and *T*_b_? Such interactions are rarely studied (Singh et al. 2020).

The “rectangular” shifts in light-dark cycles (Fig. 4A) in laboratory studies typically ignore twilight (Bradshaw & Phillips 1980), the length of which varies seasonally and latitudinally. Circadian responses can differ between rectangular versus twilight light schedules (Boulos & Macchi 2006), and thus might affect acclimation responses (Bradshaw & Phillips 1980).

*Best practices*.—Because photoperiod is a key cue of seasonality, seasonal laboratory experiments should adjust photoperiod. Realized photoperiods (as distinct from local photoperiods) can be measured in nature via telemetry, data loggers that are light sensitive (Davis & DeNardo 2010; Williams et al. 2016), or with time-lapse cameras (P. Zani, personal communication). Alternatively, photoperiod can be predicted via biophysical simulations (Fig. 4, Kearney & Porter 2020). Providing opportunities for animals to voluntarily adjust their exposure may be required to generate realistic acclimation responses to seasonality (Sinervo and Adolph 1989, Sinervo 1990).

#### Food

In a seasonal-acclimation experiment, individuals might be maintained in the lab for months at a time. Should they be fed? If so, what (type, quality), how much, and how often? For acclimation studies involving the activity seasons (e.g., spring versus summer), food should be generally provided, as animals in nature will usually be feeding in these seasons. However, some animals have empty stomachs even in activity seasons (Huey et al. 2001; Vinson & Angradi 2011), and the amount of food consumed per meal and the interval between meals may be quite variable between seasons (Christel et al. 2007). In contrast, laboratory feeding regimes are typically ad libitum or fixed ration (Table S1).

Whether food should be provided during those seasonal treatments associated with reduced or even no activity (e.g., winter dormancy) is unclear. Anorexia is a normal seasonal behavior in diverse fish, reptiles, birds, and mammals, often associated with incubation, brooding, or dormancy (Mrosovsky & Sherry 1980). The extent to which food (amount, type, quality) during winter alters acclimation responses in the laboratory is largely unresolved.

Natural history observations can indicate whether animals are feeding in winter (Nagy 1983; Filatova et al. 2019; Huey, Miles, et al. 2021; Nespolo et al. 2022) and whether feeding varies geographically. For example, the lizard *Uta stansburiana* emerges and feeds on warm winter days in California (B. Sinervo, personal communication) but not eastern Oregon (P. Zani, personal communication).

In vertebrate ectotherms, digestion and the motivation to feed can require high temperatures (Kingsolver & Woods 1997; Angilletta 2001). Thus, constant low temperatures associated with cool acclimation treatments will potentially slow and potentially stop digestion, perhaps pathologically so (Regal 1966).

Traditional acclimation experiments use the same food type, independent of season. But diet often changes seasonally (Hardison et al. 2021), either because of availability or choice. Some mammals prepare for hibernation by behaviorally altering their diet. For example, chipmunks (*Eutamias amoenus*) increase their consumption of seeds (rich in polyunsaturated oils) prior to hibernation, which enables them to lower metabolic rate during torpor and may enhance survival over winter (Geiser & Kenagy 1987). Sometimes seasonal shifts in food quality are pronounced and may affect selection for life history patterns (Maciá & Bradshaw 2000) and interact with photoperiod in terminating diapause (Bradshaw 1970). Diet can modify growth responses to temperature and cold tolerance in *Drosophila* spp. (Shreve et al. 2007; Kutz et al. 2019; Jiménez-Padilla et al. 2020) and in a calanoid copepod (Malzahn et al. 2016), as well as heat tolerance in an ant (Bujan & Kaspari 2017) and life history in an insect (Ngomane et al. 2022). Responses can be complex: responses of opaleye fish (*Girella nigricans*) to temperature and diet were trait specific (Hardison et al. 2021). Interestingly, *Drosophila melanogaster* shift dietary preferences from yeast to plant lipids at low temperature, thereby altering membrane fluidity and increasing cold tolerances (Brankatschk et al. 2018). Such a shift would be blocked if flies were unable to select food. Locusts (*Chortoicetes terminifera*) shift temperatures in response to nutritional imbalance (Clissold et al. 2013) and would inadvertently experience nutritional deficits if held at fixed temperatures.

*Best practices*.—Ideally, one would adjust laboratory feeding rates and foods to match patterns in nature (cf. Bradshaw & Holzapfel 1989; Basson & Clusella-Trullas 2015, p. 873; Danks 2007), but that will usually be impractical. Moreover, seasonal dietary information is rarely available. Nevertheless, observations on chipmunks (Geiser & Kenagy 1987) and *Drosophila* (Andersen et al. 2010; Brankatschk et al. 2018) suggest that use of standard artificial diets (rabbit chow, fly medium) may sometimes (Ngomane et al. 2022)—but not always (Davies et al. 2021)—yield biased seasonal responses. Studies that examine the impact of natural shifts in diet on seasonal phenotypes are encouraged.

### “Social distancing” and “lockdown”

Terrestrial animals in seasonal acclimation experiments are often be housed individually (Table S1), sometimes with little or no physical “enrichment” (cover, rocks, sand, and plants). Such animals have restricted opportunities for movement (exercise), exploration, and conspecific interactions relative to what animals in nature will experience during the activity season (Kiester, 1979; Killen et al. 2021), and sometimes even during hibernation (e.g., ectotherms sharing hibernacula). Does movement restriction, cage “enrichment” (or especially the lack thereof), and conspecific (or even hetero-specific) isolation affect the seasonal activity responses of isolated animals (Körner et al. 2018)?

Some animals (especially invertebrates, fishes) are often acclimated in groups (Table S1), apparently for logistic reasons. Group living may be ecologically appropriate for some species, but increased conspecific interactions can trigger aggressive behaviors and stress, possibly altering seasonal physiological capacities. For example, larval crowding affects heat tolerance in *Drosophila melanogaster* (Sørensen & Loeschcke 2001).

Traditional acclimation experiments involve single species. However, incorporating multiple species acclimation regimes may sometimes be important, at least when interspecific interactions are commensal. Midges (*Metriocnemus knabi*) and mosquitos (*Wyeomyia smithii*) naturally co-exist in pitcher plants and both feed on decaying invertebrate carcasses. Interestingly, processing by midges enhances food availability (bacteria) and energy intake by mosquitoes (Heard 1994). Mosquitoes reared without midges will have different energy budgets and potentially different plasticity responses.

Movement restriction in cages (“lockdown”) likely has diverse effects on development, physiology, morphology, and behavior. Relative to endurance-trained individuals, constrained lizards (*Anolis carolinensis*) had lower muscle mass, lower hematocrits, smaller fast glycolytic muscle fibers (Riley et al. 2017), elevated immune function (females only, Husak et al. 2017), and elevated resting metabolic rate (Lailvaux et al. 2018). These lizards are ambush predators, and more actively foraging species might be even more effected by movement restriction.

Imposed restriction on voluntary movements can have unwanted consequences. After 31 generations, mice selected for high running activity ran about three times farther per day than did controls (Careau et al. 2013). When “high runner” mice were prevented from running, they showed signs of depression and withdrawal (Malisch et al. 2009; Kolb et al. 2013). Because plasticity experiments typically block animals from natural movements, behavioral “lockdown” in laboratories will potentially bias seasonal responses.

“Social distancing” and “solitary confinement” can have marked behavioral and physiological effects on animals. In a pioneering experiment, Regal (1971) found that a male lizard greatly increased its thermoregulatory behavior (and undoubtedly its *T*_b_) in response to the presence of another male. Such social effects are well known in endotherms, but have also been detected in ectotherms (Matsubara et al. 2017). When encountering socially reared lizards, isolation-reared lizards were relatively submissive and slower to attack prey (Ballen et al. 2014). Food level affected the tendency of marsupials to huddle in winter (Nespolo et al. 2022).

*Best practices*.—Whether social conditions (solitary versus grouped housing), “impoverished” cages, and physical restriction have major effects on seasonal acclimation patterns is an open question. Ideally, housing conditions should attempt to reflect patterns in nature, but those patterns sometimes show seasonal variation in nature. For example, some lizards and snakes are territorial during the activity season but nonetheless share communal hibernacula in winter. We recognize that implementing seasonally realistic housing conditions will be difficult or even impossible for most studies. An initial goal would be to evaluate whether and how housing conditions bias seasonal responses.

## Concluding remarks

We have called attention to diverse ways that traditional laboratory regimes may bias seasonal acclimation responses. Biologically realistic regimes will of course be challenging to derive and implement. Thus, an immediate goal should be to determine which complications have strong effects and thus need to be incorporated into protocols versus which are weak and can safely be ignored. In other words, the goal is to select “*methodologies that make questions answerable in practice in a world of finite resources*” (Lewontin 2000, p. 219) and that can increase the ecological relevance of acclimation experiments. But there are logistic limits to experimental biology.

Perhaps a practical way to start is to promote studies that evaluate whether traditional protocols are “good enough” or whether they bias acclimation patterns. This requires directly comparing laboratory and field results, and we highlight some examples of validation studies (Fangue & Bennett 2003; Schultz et al. 2011; MacMillan et al. 2016; Pintor et al. 2016; Filatova et al. 2019;

Toxopeus et al. 2019; Terblanche & Hoffmann 2020). Such field-lab comparisons (validations) are encouraged.

Validation studies aren't necessary if one's goal is merely to describe the phenotypic capacities of animals in different seasons. Here one can extract animals from nature at intervals and measure them promptly (Storey et al. 1988; Zani 2005; Zhang et al. 2021). Of course, animals from some seasonal retreats are inaccessible, but sampling can be facilitated by keeping animals in semi-natural enclosures (Zani 2005; Bestion et al. 2015; Nespolo et al. 2022).

When designing a laboratory experiment, a good place to start is to try to base protocols on natural history and environmental observations in the field (reviewed in Sinclair 2001; Danks 2007). Fortunately, tools for monitoring, recording, or simulating organismal temperatures (Fig. 2B, Kearney & Porter 2020) as well as of environmental microclimates are increasingly available (Judge et al. 2018; Wickert et al. 2019).

Increasingly, seasonal patterns of microclimates, body temperatures, and activity times can even be simulated via environmental databases (e.g., ERA5) and software (Kearney & Porter 2020)(Fig. 4B), even for historical periods (Kearney, Gillingham, et al. 2020; Huey, Miles, et al. 2021). Evaluations of predictions will ultimately require comparisons of simulated responses versus those of organisms in nature (Schulte et al. 2011; Terblanche & Hoffmann 2020).

Given seasonal variation exists in many environmental factors, seasonal acclimation experiments may need to manipulate more than just temperature and photoperiod (Gunderson et al. 2016; Somero et al. 2017; Terblanche & Hoffmann 2020). However, multi-factorial experiments are still uncommon (Table S1). They will always be logistically challenging, but environmental chambers that can manipulate multiple environmental factors and incorporate realistic variability (based on organismal or weather station data) are increasingly available.

Bradshaw and Holzapfel's laboratory experiments with pitcher-plant mosquitos (e.g., Bradshaw & Holzapfel 1989) serve as exemplars of achieving relatively natural conditions in the laboratory. Mosquitos were reared inside leaves of intact pitcher plants (their natural microhabitat), exposed to natural sinewave thermoperiods that appropriately lagged natural photoperiods (with transitory dusk and dawn) by several hours, and food levels adjusted appropriately.

Field enclosures can also be used for experimental manipulations. Nespolo (2022) released marsupials into semi-natural enclosures and manipulated food levels, testing a prediction that food-constrained marsupials would enter torpor more frequently than would well fed controls. They did. Some field mesocosms (“The Metatron”) are designed for natural behaviors and dispersal, as well as to enable investigator manipulation of environmental variables (Bestion et al. 2015).

A few complex laboratory facilities have been available for decades (e.g., “Biotron,” see Figs. 19, 20 in Porter et al. 1973). Some can be programmed to mimic seasonal changes in temperature, light, and food, while still allowing an animal to behave somewhat naturally, and thus adjust its own *T*_b_, realized photoperiod, and food regime.

“AnaEE France” (Analysis and Experimentation on Ecosystems) serves as a more elaborate and synthetic way of approaching ecological studies, including seasonal ones (Clobert et al. 2018). This program consists of five modules, ranging from highly controlled laboratory facilities to field mesocosms. For example, laboratory “Ecotron” mesocosms manipulate temperature (even soil gradients!), humidity, rainfall, irradiance, O_2_ and CO_2_ concentrations—all capable of dynamic as well as step changes (Verdier et al. 2014). Aquatic and terrestrial organisms can be studied, and replication is feasible. Ecological validation of such approaches can be evaluated by releasing Ecotron-acclimated animals into nature at different seasons (Loeschcke & Hoffmann 2007) and then comparing their performance, sensitivity, and survival with those of field acclimated individuals. Unfortunately, these facilities are expensive to build and maintain, and won't be accessible to most workers. Each experimental option has associated trade-offs (Clobert et al. 2018).

Even more serious challenges will face studies that are designed to tease apart potential cues and effectors (Danks 2007) that induce seasonal acclimation or those designed to evaluate the physiological shifts underlying organismal responses (Somero et al. 2017). One's personal experience and prior research (Danks 2007) can guide appropriate factorial or fractional factorial designs, constant versus random or autocorrelated fluctuating treatments, and key environmental factors to vary (e.g., temperature, photoperiod) (Bradshaw & Holzapfel 1989; Niehaus, Wilson, et al. 2012; Singh et al. 2020; Nespolo et al. 2022). However, validating (or falsifying) the ecological and physiological relevance of such choices will be challenging (Sørensen et al. 2016; Bacigalupe et al. 2018), and incorporating individual, seasonal, geographic, and interspecific variation and interactions will be daunting (Sinervo & Adolph 1994; Gilbert & Miles 2017; Terblanche & Hoffmann 2020; Messerman & Leal 2021; Seebacher & Little 2021; Winterová & Gvoždík 2021). But challenges are also opportunities.

## Supplementary Material

obac016_Supplemental_Files
