# Supplementary material for: Designing a Seasonal Acclimation Study Presents Challenges and Opportunities
Source: Integr Org Biol. 2022 Apr 28;4(1):obac016. doi: 10.1093/iob/obac016 (PMC9175191; doi:10.1093/iob/obac016)
Supplement: obac016_Supplemental_Files [file obac016_supplemental_files.docx]

**Supplemental Information for:**

**Designing a Seasonal Acclimation Study Presents Challenges and Opportunities**

**Raymond B. Huey** **and Lauren B. Buckley**

**This supplement includes:**

**Table S1**

**Associated citations**

**Script for Fig. 3 in the main ms.**

**Table S1 –** A haphazard sample of seasonal acclimation experiments. We searched Google Scholar for papers on ectothermic animals published since 2000 using the search terms: "seasonal plasticity" or "seasonal acclimation", "thermal", and "tolerance" or "preference" or "optima" or "performance".  Citations are included in the

| **species** | **type** | **shift in temp. (°C/day)** | **environ. data from nature** | **accl. temps. relevant** | **accl. length days** | **constant temp?** | **constant photoperiod?** | **adj. food?** | **social** | **multi-factor** | **reference** |
| --- | --- | --- | --- | --- | --- | --- | --- | --- | --- | --- | --- |
| *Bathygobius cocosensis* | goby | 5C/day | partial | yes | 42 | no | yes | NA | group | no | (da Silva et al. 2019) |
| *Oligocottus maculosus* | sculpin | 0.5 °C/day | yes | unclear | 20 - 26 | yes | natural | ad lib | group? | no | (Fangue et al.) |
| *Poecilia sphenops* | molly | acute | cited ref. | yes | 30 | no | no | no | group | no | (Hernández-Rodríguez  and Bückle-Ramirez 2010) |
| *Danio rerio* | zebrafish | acute | yes | yes | 56 | yes | yes | ad lib | single | yes | (Condon et al. 2010) |
| *Ichthyosaura alpestris* | newt | 2°C/wk | yes | yes | 42 | no | no | ad lib | pairs | no | (Hadamová and Gvoždík 2011) |
| *Triturus dobrogicus* | newt | 1¨C/day | NA | partial | 56 | yes | NA | 1X or 2x week | pairs | no | (Gvoždík et al. 2007) |
| *Buergeria japonica* | frog | 2°-4°C/day | yes | yes | NA | yes | no | ad lib | group | no | (Chen et al. 2001) |
| *Dasyatis sabina* | stingray | 0.5 C/day | yes | yes | 20 | constant and fluctuating | no, 12:12 | ad lib | group | no | (Fangue and Bennett 2003) |
| *Hemigrapsus nudus* | crab | acute | cited ref. | cited ref | 14 | yes | no | ad lib | group | no | (McGaw 2003) |
| *Palaemon* spp, *Crangon* sp. *Pandalus* sp. | prawn | step | vague | partial | 7 | yes | no | ad lib | group | no | (Magozzi and Calosi 2015) |
| *Crangon crangon* | shrimp | acute | cited ref. | partial | 14 | yes | no | ad lib | group | no | (Reiser et al. 2014) |
| **Table S1 continued** |  |  |  |  |  |  |  |  |  |  |  |
| **species** | **group** | **shift in temp. (°C/day)** | **environ. data from nature** | **accl. temps. relevant** | **accl. length days** | **constant temp?** | **constant photoperiod?** | **adj. food?** | **social** | **multi-factor** | **reference** |
| *Farfantepenaeus aztecus* | shrimp | 2% salinity/day; 2 °C/day | NA | unclear | 21 | yes | no, 12:12 | ad lib | 21/reservoir | yes | (Re et al. 2005) |
| *Palaemonetes varians* | shrimp | 1 °C/week | NA | unclear | unclear | yes | no, 12:12 | ad lib | unclear | no | (Ravaux et al. 2012) |
| *Acartia* | copepod | "slowly" | yes | field collected | reared at Tb | yes | no, 12:12 | ad lib | group | no | (Sasaki and Dam 2020) |
| *Drosophila* spp. (4 sp.) | fly | acute | no | unknown | reared at Tb | yes | no | ad lib | group | no | (MacLean et al. 2019) |
| [*Forficula auricularia*](https://www.sciencedirect.com/topics/biochemistry-genetics-and-molecular-biology/auricularia) | earwig | 0.5 or 0.3°C/week | no | unknown | 33, 47 | yes | no | yes | mixed | yes | (Körner et al. 2018) |
| *Cordylus oelofseni* | lizard | acute | yes, Te & Tb | yes | 14 | diel cycles | yes | yes | unstated | no | (Basson and Clusella-Trullas 2015) |
| *Thamnophis marcianus* | snake | 5 °C every 6 days | NA | unclear | complex | no | no | fasted | solo | no | (Holden et al. 2021) |
| *Chrysemys picta* | turtle | step | yes | yes | max. ~ 170 | no | dark | no food | group | no | (Costanzo et al. 2000) |

**References**

Basson CH, Clusella-Trullas S. 2015. The behavior-physiology nexus: behavioral and physiological compensation are relied on to different extents between seasons. Physiol. Biochem. Zool. 88:384-394.

Chen T-C, Kam Y-C, Lin Y-S. 2001. Thermal physiology and reproductive phenology of *Buergeria japonica* (Rhacophoridae) breeding in a stream and a geothermal hotspring in Taiwan. Zool. Sci. 18:591-596.

Condon CH, Chenoweth SF, Wilson RS. 2010. Zebrafish take their cue from temperature but not photoperiod for the seasonal plasticity of thermal performance. J. Exp. Biol. 213(21):3705-3709.

Costanzo JP, Litzgus JD, Iverson JB, Lee RE. 2000. Seasonal changes in physiology and development of cold hardiness in the hatchling painted turtle *Chrysemys picta*. J. Exp. Biol. 203(22):3459-3470.

da Silva CRB, Riginos C, Wilson RS. 2019. An intertidal fish shows thermal acclimation despite living in a rapidly fluctuating environment. Journal of Comparative Physiology B 189:385-398.

Fangue NA, Bennett WA. 2003. Thermal tolerance responses of laboratory-acclimated and seasonally acclimatized Atlantic Stingray, *Dasyatis sabina*. Copeia 2003(2):315-325, 11.

Fangue NA, Osborne EJ, Todgham AE, Schulte PM. 2011. The onset temperature of the heat-shock response and whole-organism thermal tolerance ae tghtly correlated in both laboratory-scclimated and field-acclimatized tidepool sculpins (*Oligocottus maculosus)*. Physiol. Biochem. Zool. 84:341-352.

Gvoždík L, Puky M, Šigerlpvá M. 2007. Acclimation is beneficial at extreme test temperatures in the Danube crested newt, T*riturus dobrogicus* (Caudata, Salamandridae). Biol. J. Linn. Soc. 90:627-636.

Hadamová M, Gvoždík L. 2011. Seasonal acclimation of preferred body temperatures improves the opportunity for thermoregulation in newts. Physiol. Biochem. Zool. 84:166-174.

Hernández-Rodríguez M, Bückle-Ramirez LF. 2010. Preference, tolerance and resistance responses of *Poecilia sphenops* Valenciennes, 1846 (Pisces: Poeciliidae) to thermal fluctuations. Latin American Journal of Aquatic Research 38.

Holden KG, Gangloff EJ, Gomez-Mancillas E, Hagerty K, Bronikowski AM. 2021. Surviving winter: Physiological regulation of energy balance in a temperate ectotherm entering and exiting brumation. Gen. Comp. Endocrinol. 307:113758.

Körner M, Foitzik S, Meunier J. 2018. Extended winters entail long-term costs for insect offspring reared in an overwinter burrow. J. Therm. Biol. 74:116-122.

MacLean HJ, Overgaard J, Kristensen TN, Lyster C, Hessner L, Olsvig E, Sørensen JG. 2019. Temperature preference across life stages and acclimation temperatures investigated in four species of *Drosophila*. J. Therm. Biol. 86:102428.

Magozzi S, Calosi P. 2015. Integrating metabolic performance, thermal tolerance, and plasticity enables for more accurate predictions on species vulnerability to acute and chronic effects of global warming. Global Change Biol. 21:181-194.

McGaw IJ. 2003. Behavioral thermoregulation in *Hemigrasus nudus*, the amphibious purple shore crab. Biological Bulletin 204:38-49.

Ravaux J, Léger N, Rabet N, Morini M, Zbinden M, Thatje S, Shillito B. 2012. Adaptation to thermally variable environments: capacity for acclimation of thermal limit and heat shock response in the shrimp *Palaemonetes varians*. Journal of Comparative Physiology B 182:899-907.

Re AD, Diaz F, Sierra E, Rodríguez J, Perez E. 2005. Effect of salinity and temperature on thermal tolerance of brown shrimp *Farfantepenaeus aztecus* (Ives) (Crustacea, Penaeidae). J. Therm. Biol. 30:618-622.

Reiser S, Herrmann J-P, Neudecker T, Temming A. 2014. Lower thermal capacity limits of the common brown shrimp (*Crangon crangon*, L.). Mar. Biol. 161:447-458.

Sasaki MC, Dam HG. 2020. Genetic differentiation underlies seasonal variation in thermal tolerance, body size, and plasticity in a short-lived copepod. Ecology and Evolution 10:122000-12210.

SCRIPT FOR FIGURE 3

Using NicheMapR to simulate body temperature distributions in different seasons. Data for Ford dry Lake, CA (long = -115.09763, lat = 33.6547)

Data output from:

(http://bioforecasts.science.unimelb.edu.au/app_direct/ectotherm_ncep/). ectotherm output saved. TC = body temperature

pacman::p_load(tidyverse, here, ggplot2, lubridate, patchwork)

# Import data for Ford Dry Lake (downloaded 2021-02-15)

# tmp <- read_csv(here::here("Data", "FDL_simulations", "ectotherm-5.csv"))

# data used with lower CTmin

tmp <- read_csv(here::here("data", "input_dataFDL_daylength_hact.csv"))

tmp$dates <- mdy_hm(tmp$dates)

tmp$dates <- tmp$dates - hours(8) # times are UTC so correct for time zone

tmp$month <- month(as.Date(tmp$dates))

tmp$year <- year(tmp$dates)

tmp <- tmp %>% filter(year == 2019)

# Plots

# set up activity score (active = ACT(1,2))

tmp <- tmp %>%

mutate(

act_level = case_when(

ACT < 1 ~ "inactive",

TRUE ~ "active"))

# setup separate summer and winter files

# table N of active vs. inactive. Huge shift in proportions between

win <- c(12, 1, 2)

sum <- c(6, 7, 8)

aut <- c(9,10,11)

spr <- c(3, 4, 5)

tmp <- tmp %>%

mutate(season = case_when(month %in% win ~ "winter",

month %in% spr ~ "spring",

month %in% sum ~ "summer",

month %in% aut ~ "autumn"))

winter<- tmp %>% filter(month %in% win)

summer <- tmp %>% filter(month %in% sum)

autumn <- tmp %>% filter(month %in% aut)

spring <- tmp %>% filter(month %in% spr)

table(winter$act_level)

prop.test(table(winter$act_level))

table(spring$act_level)

prop.test(table(spring$act_level))

table(summer$act_level)

prop.test(table(summer$act_level))

table(autumn$act_level)

prop.test(table(autumn$act_level))

# compute median Tb by season and by act_level

med_Tb <- tmp %>%

group_by(season, act_level) %>%

summarise(medTb = median(TC))

#Plots of summer and of winter TC by activity level, then plot together

p_sum <- ggplot(summer, aes(TC, fill = act_level)) +

xlim(0, 40) +

geom_histogram(position = "identity", bins = 40, alpha = 0.6) +

theme_classic(base_size = 14) +

scale_y_continuous(expand = c(0, 0), limits = NULL) + theme(legend.position = "none") +

labs(title = "summer", x = "") +

scale_fill_manual(values= c(active = "red", inactive = "gray30")) +

annotate("text", x = 22 , y = 200 , label = "inactive (53.5%)", colour = "gray30", fontface = "italic") +

annotate("text", x = 30 , y = 200 , label = "active", colour = "red", fontface = "italic") +

geom_segment(x = 34.9, y = 75, xend = 34.9, yend = 10, lineend = "butt", linejoin = "mitre", size = 1, arrow = arrow(length = unit(0.075, "inches")), colour = "black") +

geom_segment(x = 26.0, y = 75, xend = 26.0, yend = 10, lineend = "butt", linejoin = "round", size = 1, arrow = arrow(length = unit(0.075, "inches")), colour = "white")

p_spr <- ggplot(spring, aes(TC, fill = act_level)) +

xlim(0, 40) +

geom_histogram(position = "identity", bins = 40, alpha = 0.6) +

theme_classic(base_size = 14) +

scale_y_continuous(expand = c(0, 0), limits = NULL) + theme(legend.position = "none") +

labs(title = "spring", x = "Body temperature (°C)") +

scale_fill_manual(values= c(active = "red", inactive = "gray30")) +

theme(plot.title = element_text(colour = "gray30")) +

annotate("text", x = 10 , y = 200 , label = "inactive (38.2%)", colour = "gray30", fontface = "italic") +

annotate("text", x = 30 , y = 200 , label = "active", colour = "red", fontface = "italic") +

geom_segment(x = 34.9, y = 75, xend = 34.9, yend = 10, lineend = "butt", linejoin = "mitre", size = 1.5, arrow = arrow(length = unit(0.075, "inches")), colour = "black") +

geom_segment(x = 14.6, y = 75, xend = 14.6, yend = 10, lineend = "round", linejoin = "round", size = 1.5, arrow = arrow(length = unit(0.075, "inches")), colour = "white")

p_aut <- ggplot(autumn, aes(TC, fill = act_level)) +

xlim(0, 40) +

geom_histogram(position = "identity", bins = 40, alpha = 0.6) +

theme_classic(base_size = 14) +

scale_y_continuous(expand = c(0, 0), limits = c(0, 500)) + theme(legend.position = "none") +

labs(title = "autumn", x = "Body temperature (°C)") +

scale_fill_manual(values= c(active = "red", inactive = "gray30")) +

theme(plot.title = element_text(colour = "gray30")) +

annotate("text", x = 15 , y = 200 , label = "inactive (65.7%)", colour = "gray30", fontface = "italic") +

annotate("text", x = 30 , y = 200 , label = "active", colour = "red", fontface = "italic") +

geom_segment(x = 34.6, y = 75, xend = 34.6, yend = 10, lineend = "butt", linejoin = "mitre", size = 1.5, arrow = arrow(length = unit(0.075, "inches")), colour = "black") +

geom_segment(x = 17.8, y = 75, xend = 17.80, yend = 10, lineend = "butt", linejoin = "mitre", size = 1.5, arrow = arrow(length = unit(0.075, "inches")), colour = "white")

p_win <- ggplot(winter, aes(TC, fill = act_level)) +

xlim(0, 40) +

geom_histogram(position = "identity", bins = 40, alpha = 0.6) +

theme_classic(base_size = 14) +

scale_y_continuous(expand = c(0, 0), limits = c(0, 500)) + theme(legend.position = "none") +

labs(title = "winter", x = "Body temperature (°C)") +

scale_fill_manual(values= c(active = "red", inactive = "gray30")) +

theme(plot.title = element_text(colour = "gray30")) +

annotate("text", x = 2.5 , y = 200 , label = "inactive (94.7%)", colour = "gray30", fontface = "italic") +

annotate("text", x = 30 , y = 200 , label = "active", colour = "red", fontface = "italic") +

geom_segment(x = 32.3, y = 75, xend = 32.3, yend = 10, lineend = "butt", linejoin = "mitre", size = 1.5, arrow = arrow(length = unit(0.075, "inches")), colour = "black") +

geom_segment(x = 8.4, y = 75, xend = 8.40, yend = 10, lineend = "butt", linejoin = "mitre", size = 1.5, arrow = arrow(length = unit(0.075, "inches")), colour = "white")

# combine plots (patchwork)

p_spr / p_sum / p_aut / p_win

SCRIPT FOR FIGURE 4B

# Using NicheMapR to simulate hours of activity of a lizard over the year at Ford Dry Lake, CA long = -115.09763, lat = 33.6547, (downloaded 2021-02-28)

# (http://bioforecasts.science.unimelb.edu.au/app_direct/ectotherm_ncep/). I saved the ectotherm output = input_data-2021-02-28.csv, TC = body temperature.

tmp <- read_csv(here::here("Data/FDL_simulations", "ectotherm-2021-02-28.csv"))

tmp$dates <- mdy_hm(tmp$dates)

tmp$dates<- tmp$dates - hours(8) # times are UTC so correct for time zone

tmp$month <- month(as.Date(tmp$dates))

tmp$year <- year(tmp$dates)

tmp <- tmp %>% filter(year == 2019)

tmp <- tmp %>% mutate(week = week(dates))

tmp <- tmp %>% mutate(

act_level = case_when(

ACT < 1 ~ "inactive",

TRUE ~ "active")) # active (ACT = 1, 2) vs inactive(ACT = 0)

tmpPP <- tmp %>% # compute average h.act per week

group_by(week) %>%

mutate(hact = sum(act_level == "active")/7)

summary(tmpPP$hact) # exposure over year

summary(tmpPP$DAYLENGTH) # daylength over year

summary(tmpPP$hact[tmp$month %in% c(12, 1, 2)]) # exposure in winter

summary(tmpPP$DAYLENGTH[tmp$month %in% c(12, 1, 2)]) # daylength in winter

summary(tmpPP$hact[tmp$month %in% c(6, 7, 8)]) # exposure in summer

summary(tmpPP$DAYLENGTH[tmp$month %in% c(6, 7, 8)]) # daylength in summer

```{r plot}

photo_plot <- ggplot(tmpPP) +

scale_y_continuous(expand = c(0, 0), limits = c(0, 15)) +

geom_line(aes(dates, DAYLENGTH), color = "red") +

geom_line(aes(dates, hact)) +

labs(title = "Photoperiod & exposure time, Ford Dry Lake, CA",

y = "Daylength (h) and \n hours of exposure (averaged by week)",

x = "") +

theme_minimal_hgrid(10) +

annotate("text", x =tmpPP$dates[300], y = 10.7, label = "daylength", col = "red", size = 3) +

annotate("text", x =tmpPP$dates[2436], y = 3.5, label = "exposure time", size = 3)
